# Molecular profiling of cervical cancer progression

**DOI:** 10.1038/sj.bjc.6603543

**Published:** 2007-01-23

**Authors:** T Hagemann, T Bozanovic, S Hooper, A Ljubic, V I F Slettenaar, J L Wilson, N Singh, S A Gayther, J H Shepherd, P O A Van Trappen

**Affiliations:** 1 Centre for Translational Oncology, Institute of Cancer and the CR-UK Clinical Centre, Barts and The London, Queen Mary's School of Medicine and Dentistry, London, UK; 2 Department of Obstetrics and Gynaecology, Clinical Centre of Belgrade, Belgrade, Serbia-Montenegro, UK; 3 Department of Histopathology, St Bartholomew's Hospital, London, UK; 4 Translational Research Laboratories, Department of Gynaecological Oncology, University College London, London, UK; 5 Gynaecological Cancer Centre, St. Bartholomew's Hospital, London, UK

**Keywords:** molecular profiling, cervical cancer, lymph node, micrometastases, recurrent tumours

## Abstract

Most cancer patients die of metastatic or recurrent disease, hence the importance to identify target genes upregulated in these lesions. Although a variety of gene signatures associated with metastasis or poor prognosis have been identified in various cancer types, it remains a critical problem to identify key genes as candidate therapeutic targets in metastatic or recurrent cancer. The aim of our study was to identify genes consistently upregulated in both lymph node micrometastases and recurrent tumours compared to matched primary tumours in human cervical cancer. Taqman Low-Density Arrays were used to analyse matched tumour samples, obtained after laser-capture microdissection of tumour cell islands for the expression of 96 genes known to be involved in tumour progression. Immunohistochemistry was performed for a panel of up- and downregulated genes. In lymph node micrometastases, most genes were downregulated or showed expressions equal to the levels found in primary tumours. In more than 50% of lymph node micrometastases studied, eight genes (AKT, BCL2, CSFR1, EGFR1, FGF1, MMP3, MMP9 and TGF-*β*) were upregulated at least two-fold. Some of these genes (AKT and MMP3) are key regulators of epithelial–mesenchymal transition in cancer. In recurrent tumours, almost all genes were upregulated when compared to the expression profiles of the matched primary tumours, possibly reflecting their aggressive biological behaviour. The two genes showing a consistent downregulated expression in almost all lymph node metastases and recurrent tumours were BAX and APC. As treatment strategies are very limited for metastatic and recurrent cervical cancer, the upregulated genes identified in this study are potential targets for new molecular treatment strategies in metastatic or recurrent cervical cancer.

Cervical cancer is one of the leading causes of death in women worldwide, especially in developing countries owing to the lack of adequate screening programmes. The causative agent of cervical carcinogenesis is human papillomavirus (HPV), with insertion of viral oncogenes into the host DNA (Walboomers *et al*, 1999). More than 100 different HPV types have been identified and those associated with cervical cancer are subdivided as high-risk types, such as HPV16 and HPV18 (zur Hausen, 2000, 2002). However, the molecular mechanisms controlling cervical cancer progression are poorly understood.

Meta-analysis of DNA microarray data in cancer has revealed that hundreds of genes are differentially expressed between cancer tissues, including cervical cancer and their normal counterparts (Shim *et al*, 1998; Rubin *et al*, 2004; Segal *et al*, 2004; Hudelist *et al*, 2005). It became evident that many of these genes are epiphenomena and that only a subset of genes is associated with tumour progression and metastasis (Ramaswamy *et al*, 2003; Roepman *et al*, 2005). In cervical cancer, gene expression profiling could be a useful tool for molecular classification and prediction of treatment response, but large studies are lacking (Wong *et al*, 2003). A 17-gene signature has been associated with metastasis in a variety of human solid tumours, including lung, breast, prostate, colorectal, uterine and ovarian cancer (Ramaswamy *et al*, 2003). However, none of these genes represented markers of metastasis, and the signature reflected the gene expression profile of both neoplastic and stromal cells. To identify key genes involved in a particular cancer progression model it is essential to analyse matched tumour samples of different stages. These studies could direct new therapeutic strategies targeting specific candidate genes. Although a variety of genes have been implicated in cervical cancer invasion, migration and lymph node metastasis, most studies have used only cervical cancer cell lines or primary tumours (Shim *et al*, 1998; Ruutu *et al*, 2002; Van Trappen *et al*, 2002; Vazquez-Ortiz *et al*, 2005a). To our knowledge, no data are available on gene expression profile differences between primary tumours and matched metastatic or recurrent tumours in cervical cancer.

In this report, we attempt to identify genes up- or downregulated in lymph node micrometastases and recurrent tumours when compared with the matched primary tumours. To explore the gene expression profile of cervical cancer cells we used laser-capture microdissection to extract tumour cell islands. Taqman Low-Density Array, based on quantitative real-time RT–PCR, was used to screen matched tumour samples for 96 selected genes involved in several molecular processes such as angiogenesis, matrix degradation, cell cycle, oncogenic pathways, DNA repair mechanisms, adhesion, invasion, migration, cell proliferation and apoptosis. Immunohistochemistry was performed for several up- and downregulated genes.

## MATERIALS AND METHODS

### Tissue specimens

Tumour specimens were obtained from cervical cancer patients who underwent a radical hysterectomy and bilateral pelvic lymph node (LN) dissection for early-stage cervical cancer International Federation of Gynecology and Obstetrics (FIGO)-stage I–II at the Gynecological Cancer Centre of St Bartholomew's Hospital and the Department of Obstetrics and Gynecology of the Clinical Centre of Serbia between January 1999 and December 2002. From those patients, surgical specimens were formalin fixed immediately after removal and archival paraffin-embedded tissue was retrieved of following tissues, primary tumours, paired lymph nodes with micrometastasis or recurrent tumours where available. Patients were staged according to the guidelines of the FIGO. All specimens were reviewed by a pathologist (NS) of the Histopathology Department at St Bartholomew's Hospital and standard clinicopathological features (grade, histological subtype and lymph node metastasis) were recorded. All primary tumour samples included in the study were squamous cell carcinomas (SCC) with paired lymph node micrometastases (between 200 and 2000 *μ*m according to TNM classification) or recurrent tumours. The study was approved by the East London and the City Local Research Ethics Committee (T/02/046).

### Microdissection, RNA extraction and RT–PCR

Before microdissection P.A.L.M slides (1 mm pen slides, P.A.L.M. Microlaser Technologies AG, Bernried, Germany) were first treated with 1 × RNAse Zap treatment for 2 min and then washed three times in DEPC water. After drying, the slides were sterilised in UV light for 30 min in a UV stratalinker (Stratagene, La Jolla, CA, USA), membrane face up. Serial 7-*μ*m paraffin sections were cut with a fresh blade, mounted on prepared slides and incubated at RT for 30 min to achieve optimal tissue adhesion to the membrane. All staining baths were washed with RNAse Zap before use. Deparaffinisation was carried out by incubation in xylene (2 × 2 min), followed by washing and rehydration in ethanol (100% ethanol 30 s, 95% ethanol 30 s, 70% ethanol 30 s and H_2_O 10 s rinse). Sections were stained with haematoxylin for 1 min and washed twice for 1 min in H_2_O. Dehydration followed, in ethanol (70% ethanol 30 s, 95% ethanol 2 × 30 s, 100% ethanol 1 min). Slides were then ready after drying for processing by laser-assisted microdissection (LAMD).

For haematoxilyin and eosin staining, sections were processed as mentioned above. After dehydration in 70% ethanol for 30 s, slides were stained for 5 s in eosin, then rinsed shortly in water. The dehydration was completed as mentioned above.

Microdissection of the membrane-mounted sections (P.A.L.M.) was performed with a high-resolution nitrogen UV laser (P.A.L.M. MicroLaser System, Zeiss, Oberkochen, Germany). In brief, RNA was extracted from laser microdissected tissue samples using the Ambion paraffin block RNA isolation kit (Ambion, Austin, TX, USA) according to the manufacturer's instructions. After the cutting event, the same laser was used to catapult the dissected material into a microtube cap containing 100 *μ*l Proteinase K digestion buffer (5 *μ*l proteinase K), incubated for 1 h at 45°C and inactivated at 95°C for 10 min. A 600 *μ*l volume of RNA extraction buffer was added and the sample was incubated for 5 min at RT. The sample was extracted using phenol : chloroform. The aqueous layer was transferred to a fresh tube and mixed with 2 *μ*l linear acrylamide. Isopropanol (1 : 1) precipitation was carried out overnight. Following washing in 75% ethanol, the sample was resuspended in 17 *μ*l H_2_O. The RNA was DNase I (2 U *μ*l^−1^) treated for 15 min at 37°C.

### Taqman Low-Density Arrays

Taqman Low-Density Arrays (Part No. 4342261, Applied BioSystems, 850 Lincoln Centre Drive, Foster City, CA, USA) were used for gene expression profiling based on real-time quantitative RT–PCR to compare primary tumour and lymph node or recurrence tissue. Briefly, 2 *μ*g DNase-treated RNA from each sample was used for reverse transcription into 100 *μ*l cDNA. We used Random Hexamers (Promega, Madison, WI, USA) and M-MLV RT enzyme (Roche Diagnostics, Mannheim, Germany) to synthesise cDNA. For each sample, 40 *μ*l of cDNA synthesised above were then mixed with 210 *μ*l Taqman universal PCR master mix (PE Applied Biosystems, New Jersey, NJ, USA) and 170 *μ*l PCR water to form reaction mix. A 400 *μ*l portion of this reaction mix was then put on microfluidic card into 192 mini wells containing primers and probes of 96 genes in duplicate. These 96 genes, known to be involved in tumour progression, included DNA damage repair genes, cell cycle control genes, common oncogenes, tumour suppressor genes and metastasis-associated genes. Human 18S and GAPDH were used as endogenous controls. For each 384 well card, two cDNA samples (matched) were included at the same time for real-time RT–PCR reaction and analysis. The real-time RT–PCR reaction and laser scanning was performed on ABI 7900HT genotyper with SDS2.1 software. The expression level of each gene is analysed on the mean of its duplicates. Only the genes with reproducible amplification curves of both duplicates were analysed and presented.

### Immunohistochemistry

Immunohistochemical staining was performed for candidate genes upregulated at mRNA level. Primary human monoclonal antibodies to EGF-R (clone 1F4, Cell Signaling Technology, Inc. 3 Trask Lane, Danvers, MA, USA), AKT, FGF1, BCL2, CSF1R, MMP3 and MMP9 (R&D systems, Abingdon, UK), Her2/erbB2 (Cell Signaling Technology, USA), phospho-Her2/erbB2 (Tyr1221/1222, Cell Signaling Technology, USA) were used for staining. Briefly, slides were blocked with horse serum (5 min), incubated in a humid chamber for 60 min with primary antibody, and for 30 min each with biotinylated secondary antibody and avidin-biotinylated horseradish peroxidase complex (ABC). Endogenous peroxidase activity was blocked by 0.5% hydrogen peroxide (H_2_O_2_) in methanol for 15 min. Washing was carried out using PBS buffer. Colour development was obtained with 3,3-diaminobenzidine-tetrahydrochloride for 10 min. Sections were counterstained in toluidine blue for 4 min, washed for 4 min in water, and dried in ethanol. Sections were evaluated by quantitative analysis, the number of immunohistochemical (IHC)-positive tumour cells were calculated on the total number of tumour cells (percentage) in three high-power fields per section. The median was calculated for both primary tumours and metastases/recurrent tumours and statistical differences were analysed using the Fisher's Exact test.

## RESULTS

**Figure 1:**
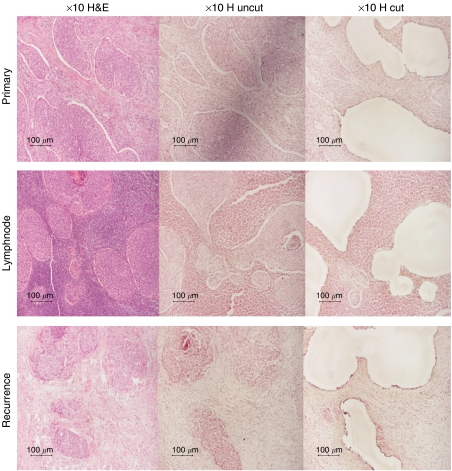
Slides of primary cervical cancers, lymph node micrometastases and recurrent tumours for haematoxylin and eosin (H&E) staining (first column; 4 *μ*m section) and haematoxylin (H) staining before (second column; 7 *μ*m section) and after microdissection (third column; 7 *μ*m section).

**Figure 2:**
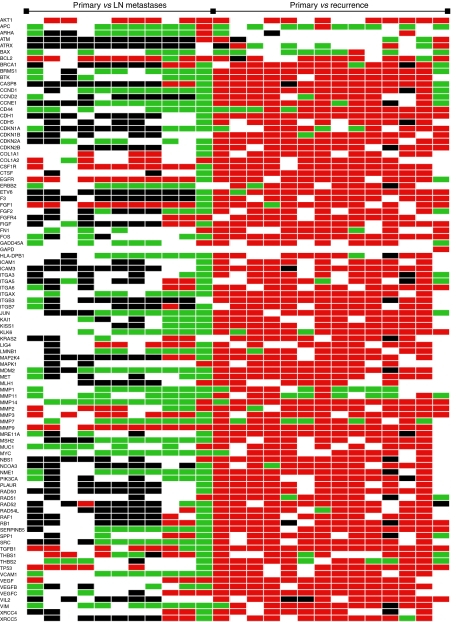
Genes differentially expressed between LN micrometastases or recurrent tumours and matched primary cervical cancers. The figure shows genes upregulated (red) or downregulated (green) (at least two-fold) in LNM (*n*=11) and RT (*n*=14) respectively, when compared with the expression profiles found in primary tumours. Genes that did not vary in their expression level are shown in white, and genes with unsatisfactory results (i.e. aberrant amplification curves or no expression) were labelled black.

We analysed 25 paired tumour samples for gene expression profile differences: 11 primary cervical tumours with matched lymph node micrometastases and 14 primary cervical tumours with matched recurrent tumours. Laser-assisted microdissection was used to extract tumour cell islands from those samples. The appearance of tissue before and after microdissection is illustrated in Figure 1. RNA was extracted and subsequent cDNA was analysed using Taqman Low-Density Arrays. A summary of the genes whose expression was altered consistently in replicate experiments is presented in Figure 2. The figure shows genes upregulated (red) or downregulated (green) (at least 2-fold) in the lymph node micrometastases (*n*=11) and recurrent tumours (*n*=14) respectively, when compared with the expression profiles found in primary cervical tumours. Genes that did not vary in their expression level are shown in white and genes with unsatisfactory results (i.e. aberrant amplification curves or no expression) were labelled black. The housekeeping gene GAPDH showed identical expression levels in all matched samples analysed apart from one.

In lymph node micrometastases, most genes were downregulated or showed equal expression when compared with the expression levels in primary cervical tumours. In more than half (>6/11) of these, there were eight genes consistently >2-fold upregulated in lymph node micrometastases when compared with primary tumours. These eight genes were AKT, BCL2, CSFR1, EGFR1, FGF1, MMP3, MMP9 and transforming growth factor (TGF)-*β*. Twenty-five genes were consistently <2-fold downregulated (Table 1).

In recurrent tumours, almost all genes were upregulated (>2-fold), possibly reflecting their aggressive biological behaviour. In total, 88 of 96 genes were upregulated (including the eight genes commonly upregulated in lymph node micrometastases) in more than 50% (>7/14) of recurrent cases. The two genes showing a consistent pattern of downregulated expression in both lymph node micrometastases and recurrent tumours were BAX and APC.

### Immunohistochemical staining

Immunohistochemical (IHC) staining was performed for both up- and downregulated gene(s) in all LN micrometastases (*n*=11) and compared to the staining results in the primary tumours. Quantitative analysis was performed by calculating the number of IHC-positive tumour cells on the total number of tumour cells (percentage) in three high power fields per section. The median was calculated for both primary tumours and lymph node micrometastases and statistical differences were analysed using the Fisher's Exact test.

**Figure 3:**
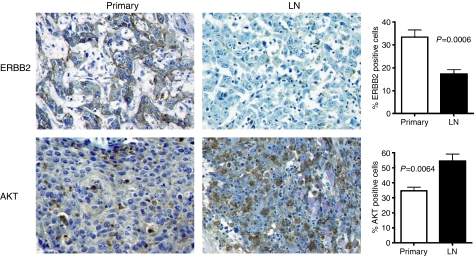
Immunohistochemistry (IHC) for ERBB2 and AKT in matched primary cervical tumours and LN micrometastases. Quantitative analysis of IHC-positive cells revealed that ERBB2 showed a significant stronger expression in the primary tumour (*P*=0.0006), whereas AKT showed a significant stronger expression in the LN metastases (*P*=0.0064).

AKT was significantly upregulated in lymph node micrometasases when compared with the primary tumour (*P*=0.0064; Figure 3). Similarly, immunohistochemistry also confirmed upregulation of CSF1R and EGFR in lymph node micrometastases (data not shown). We could also confirm significant downregulation of ERBB2 (*P*=0.0006; Figure 3). Immunohistochemical differences for MMP3, MMP9, FGF1 and BCL2 were inconclusive (data not shown). The reason therefore could either be the tissue embedding technique or that there is still a post-transcriptional modification of these genes.

## DISCUSSION

In this study, we have investigated differential gene expression profiles between LN micrometastases or recurrent tumours and matched primary cervical cancers. We used a unique set of cervical cancer samples from patients who underwent a radical hysterectomy and pelvic lymph node dissection for presumed early-stage cervical cancer. However, these patients demonstrated on histological examination small clusters of LN metastases (micrometastases), which were not identifiable on preoperative investigations (imaging) or during surgery. Cervical cancers with LN metastases are normally treated by primary chemo-radiotherapy, hence the unique set of matched primary tumours and LN micrometastases obtained for this study.

In order to obtain expression profiles of tumour cell islands only we used LAMD of paraffin-embedded tissue and microfluidic cards based on real-time quantitative RT–PCR, a technique similar to that described previously (Specht *et al*, 2001). This method was used, as LN micrometastases can only be identified after histological examination of paraffin-embedded tissue, and fresh frozen human samples of LN micrometastases are difficult, if not impossible to obtain. The microfluidic card contained 96 genes previously described in tumour progression or identified as markers of metastasis or clinical outcome (van ‘t Veer *et al*, 2002; Ramaswamy *et al*, 2003; Rhodes and Chinnaiyan, 2004; Segal *et al*, 2004; Roepman *et al*, 2005). Data obtained from microarrays of RNA retrieved from paraffin-embedded tissue are not reliable hence our application of microfluidic cards with selected genes. Although microarrays could give a broader signature we aimed at identifying a selective gene signature both in LN micrometastases and recurrent tumours to identify markers as potential therapeutic targets. The 96 selected genes are involved in several molecular processes such as angiogenesis, matrix degradation, cell cycle, oncogenic pathways, DNA repair mechanisms, adhesion, invasion, migration, cell proliferation and apoptosis. In LN metastases, most genes were downregulated or showed equal expression with the levels found in primary tumours, possibly reflecting their motility behaviour requiring (temporarily) less molecular pathways compared with primary tumours. In more than 50% of LN metastases, eight genes were upregulated at least two-fold and 25 genes were downregulated at least two-fold. In recurrent tumours, almost all genes were upregulated at least two-fold, possibly reflecting their aggressive biological behaviour. The two genes showing a consistent pattern of downregulated expression in both LN metastases and recurrent tumours were BAX and APC. The functional role of both genes has been well described in cancer, with the BCL2 homologue gene BAX playing a key role in tumour apoptosis as a proapoptotic gene and APC acting as a tumour suppressor gene, which is frequently inactivated in cervical cancer by hypermethylation (LeBlanc *et al*, 2002; Reesink-Peters *et al*, 2004; Soufla *et al*, 2005).

Here we describe eight genes (AKT, BCL2, CSFR1, EGFR1, FGF1, MMP3, MMP9 and TGF-*β*) found to be commonly upregulated in LN micrometastases in cervical cancer, as well as their functional role previously described in cancer cell lines. These genes were also upregulated in almost all recurrent tumours analysed making them interesting targets for therapeutic strategies. AKT is a serine/threonine kinase acting as a signal transduction protein and downstream mediator of phosphatidylinositol-3-kinase (PI3K) that plays a central role in tumour progression and influences prognosis in several cancers (Lim *et al*, 2005; Tang *et al*, 2006). The oncogenic pathway PI3K/AKT plays a central role in epithelial–mesenchymal transition, a critical feature in cancer invasion and metastasis by reducing intercellular adhesion and increasing cancer cell motility (Larue and Bellacosa, 2005). Upregulation of AKT in LN metastases in this study suggests its potential role in epithelial–mesenchymal transition of cervical cancer cells during metastasis. In the cervical cancer cell line C33A, the PI3K/AKT pathway has a key role in cancer progression towards a metastatic phenotype (Mora *et al*, 2006). BCL2 is an antiapoptotic gene and increased expression is associated with poor responses to systemic treatment of cancer (Andersen *et al*, 2005; Kausch *et al*, 2005). Colony stimulating factor-1 receptor (CSFR1) is involved in tumour cell invasion and migration, and high level expression is associated with poor prognosis in cancer patients (Tsuzuki *et al*, 2005). In various cervical cancer cell lines known to exhibit different degrees of aggressivity, the highest levels of colony stimulating factor are found in the most aggressive cell lines (Bretscher *et al*, 2000). The CSF1 produced by cancer cells promotes the expression of EGF by macrophages (Goswami *et al*, 2005). In addition, EGF promotes the expression of CSF-1 by cancer cells, thereby generating a positive feedback loop. Disruption of this loop by blockade of either EGF receptor or CSF-1 receptor signalling is sufficient to inhibit both macrophage and tumour cell migration and invasion. Epidermal growth factor receptor (EGFR) is highly expressed in many tumours, including those of the cervix, and there is a clear relationship between HPV E6/E7 oncoproteins and EGFR function (Hu *et al*, 1997). Blockade of EGFR in cervical cancer cell lines induces increased expression of genes that stimulate apoptosis and suppresses experimental metastasis (Kim *et al*, 2004; Woodworth *et al*, 2005). Fibroblast growth factor 1 (FGF1) is a growth factor for both tumour and stromal (endothelial) cells and induces the expression of matrix metalloproteinases (MMPs) and angiogenesis in cancer, hence its involvement in tumour progression (Aonuma *et al*, 1998; Kwabi-Addo *et al*, 2004; Udayakumar *et al*, 2004). The MMPs, a family of proteolytic enzymes that degrade different components of the extracellular matrix, play important roles in the different stages of tumour progression. Matrix metalloproteinase 3 (stromelysin 1) plays an important role in breast cancer progression, and it has shown to have increased levels, as well as activity, in breast cancer brain metastasis in a rat model (Mendes *et al*, 2005). Similar to AKT, MMP3 can also cause epithelial–mesenchymal transition in cancer (Radisky *et al*, 2005). Matrix metalloproteinase 9 plays a central role in connective tissue degradation, tumour-induced angiogenesis, cell proliferation/apoptosis and cell migration in various tumour types including cervical cancer (van Kempen and Coussens, 2002; Van Trappen *et al*, 2002; Zucker and Vacirca, 2004; Vazquez-Ortiz *et al*, 2005b). Recently, one study has shown that MMP9 forms a complex with the hyaluronan receptor CD44 on the surface of breast cancer cells and activates downstream TGF-*β*, facilitating tumour cell survival, invasion and metastasis (Yu and Stamenkovic, 2004). Similar mechanism(s) could occur at the level of tumour cell survival of LN micrometastases in cervical cancer. Transforming growth factor (TGF)-*β* stimulates epithelial–mesenchymal transition of SiHa cervical cancer cells, indicating a positive role in the invasive transition of cervical cancer (Yi *et al*, 2002). Although TGF-*β* is both a tumour suppressor and tumour promoter, TGF-*β* inhibitors are currently widely tested for the treatment of cancer (Muraoka-Cook *et al*, 2004; Elliott and Blobe, 2005; Lahn *et al*, 2005). Various genes were downregulated in LN micrometastases, such as major histocompatibility complex (MHC) class II antigens and NME1. Underexpression of MHC molecules involved in T-lymphocyte-mediated tumour cell recognition may be one important escape mechanism used by metastatic cancer cells, and these cells can be ignored by the immune system for a long time despite the presence of immunocompetent cells (Pantel *et al*, 1991). NME1, a metastasis-suppressor gene, shows reduced expression in highly metastatic breast cancer cells and was downregulated in cervical cancer LN metastases in this study (Rubio *et al*, 2006).

Although several cancer gene signatures have shown prognostic significance in a variety of cancers, it remains a critical problem to identify key genes as candidate therapeutic targets. As cancer cells require the expression of different genes along the different stages of tumour progression, it is essential to obtain expression profile data from the different steps of tumour progression. In this study, we have identified a panel of tumour-associated genes upregulated in LN micrometastases, as well as in recurrent tumours which could be potential therapeutic targets in metastatic or recurrent cervical cancer.

## Figures and Tables

**Table 1:** Genes differentially expressed in lymph node micrometastases when compared with matched primary cervical cancers

**Gene**	**Gene name**	**ID**
*Upregulated in LN metastases*		
AKT	v-akt murine thymoma viral oncogenes homologue 1	Hs00178289_m1
BCL2	B-cell CLL/lymphoma 2	Hs00153350_m1
CSFR1	Colony-stimulating factor 1 receptor	Hs00234617_m1
EGFR1	Epidermal growth factor receptor	Hs00193306_m1
FGF1	Fibroblast growth factor 1 (acidic)	Hs00265254_m1
MMP3	Matrix metalloproteinase 3 (stromelysin 1, progelatinase)	Hs00233962_m1
MMP9	Matrix metalloproteinase 9 (gelatinase B, 92 kDa gelatinase)	Hs00234579_m1
TGFB	Transforming growth factor, beta 1	Hs00171257_m1
		
*Downregulated in LN metastases*		
APC	*Adenomatosis polyposis coli*	Hs00181051_m1
ARHA	Ras homologue gene family, member A	Hs00357608_m1
BRMS1	Breast cancer metastasissuppressor 1 (Interim)	Hs00363036_m1
CCND1	Cyclin D1 (PRAD1: parathyroid adenomatosis 1)	Hs00211039_m1
CCNE1	Cyclin E1	Hs00233356_m1
CD44	CD44 antigen (homing function and Indian blood group system)	Hs00153310_m1
ERBB2	v-erb-b2 erythroblastic leukaemia viral oncogenes homologue 2	Hs00170433_m1
HLA-DPB1	Major histocompatibility complex, class II, DP beta 1	Hs00157955_m1
ITGAX	Integrin, alpha X (antigen CD11C (p150), alpha polypeptide)	Hs00174217_m1
JUN	v-jun sarcoma virus 17 oncogene homologue (avian))	Hs00277190_m1
KLK6	Kallikrein 6 (neurosin, zyme)	Hs00160519_m1
LMNB1	Lamin B1	Hs00194369_m1
MDM2	Mdm2, transformed 3T3 cell double minute 2, p53-binding protein	Hs00242813_m1
MMP1	Matrix metalloproteinase 1 (interstitial collagenase)	Hs00233958_m1
MMP7	Matrix metalloproteinase 7 (matrilysin, uterine)	Hs00159163_m1
MMP11	Matrix metalloproteinase 11 (stromelysin 3)	Hs00171829_m1
MMP14	Matrix metalloproteinase 14 (membrane-inserted)	Hs00237119_m1
MSH2	mutS homologue 2, colon cancer, nonpolyposis type 1 (*Escherichia. coli*)	Hs00179887_m1
MUC1	Mucin 1, transmembrane	Hs00410317_m1
MYC	v-myc myelocytomatosis viral oncogenes homologue (avian)	Hs00153408_m1
NME1	Nonmetastatic cells 1, protein (NM23A) expressed in	Hs00264824_m1
SERPINB5	Serine (or cysteine) proteinase inhibitor, clade B (ovalbumin), member5	Hs00184728_m1
SRC	v-src sarcoma (Schmidt-Ruppin A-2) viral oncogene homologue (avian)	Hs00178494_m1
VCAM1	Vascular cell adhesion molecule 1	Hs00174239_m1
VIM	Vimentin	Hs00185584_m1
